# Peripartum Respiratory Failure in a Patient With Severe Preeclampsia

**DOI:** 10.7759/cureus.79832

**Published:** 2025-02-28

**Authors:** Maria Ntioudi, Sophia Gelatou, Victoria Karypidou, Dimitra Alatzidou, Thomas Karagkiouzis

**Affiliations:** 1 Department of Obstetrics and Gynecology, General Hospital of Giannitsa, Giannitsa, GRC; 2 Department of Anesthesiology, General Hospital of Giannitsa, Giannitsa, GRC; 3 Intensive Care Unit, General Hospital of Giannitsa, Giannitsa, GRC

**Keywords:** general anesthesia, gestational hypertension, preeclampsia, preeclampsia complications, respiratory failure, severe preeclampsia, severe respiratory failure

## Abstract

Preeclampsia is a major hypertensive disorder of pregnancy associated with increased maternal, fetal, and neonatal risks. Acute respiratory failure, though rare, is a severe complication that may arise due to preeclampsia or unrelated medical conditions. We present a case of a 23-year-old primiparous woman admitted to our Emergency Department with gestational hypertension during her third trimester. She was hospitalized for blood pressure regulation and preeclampsia screening, which included urine protein analysis, blood tests for liver and kidney function, and fetal monitoring through cardiotocography. Laboratory tests revealed albuminuria but no indicators of severe preeclampsia. On the fourth day of hospitalization, despite antihypertensive treatment, the patient’s blood pressure remained elevated, and she developed severe headaches. An emergency caesarean section was performed. During the procedure, she experienced severe bronchospasm, causing transient oxygen desaturation. Despite initial improvement, persistent hypoxemia required increased oxygen administration. Imaging and cardiological evaluations ruled out major complications, and she was admitted to the intensive care unit for eight days. Following a 14-day hospitalization, the patient was discharged in stable hemodynamic and respiratory condition, with follow-up instructions for cardiology and pulmonology assessment. Severe preeclampsia can lead to life-threatening complications, including acute respiratory failure. Prompt differential diagnosis and timely intervention are crucial to improving maternal outcomes.

## Introduction

Hypertensive disorders of pregnancy are among the leading causes of maternal and neonatal morbidity and mortality worldwide. Preeclampsia affects approximately 2% to 8% of pregnancies and is characterized by new-onset hypertension and proteinuria, sometimes accompanied by organ dysfunction [1]. The pathophysiology of preeclampsia is complex, involving abnormal placentation, endothelial dysfunction, and systemic inflammation [2]. In more detail, preeclampsia is a complex disorder characterized by widespread endothelial dysfunction, primarily driven by abnormal placental development and maternal vascular maladaptation. A crucial event in its pathogenesis is the incomplete trophoblastic invasion of the maternal spiral arteries, leading to insufficient remodeling of these vessels. As a result, placental perfusion remains compromised due to persistent high-resistance and low-capacitance blood flow, creating a state of relative ischemia. This ischemic environment is believed to trigger the release of circulating factors that induce systemic endothelial dysfunction. Subsequently, endothelial injury contributes to the activation of the coagulation cascade, impaired vasodilation, increased vascular permeability, and tissue hypoxia. These mechanisms collectively give rise to the hallmark clinical signs and symptoms of preeclampsia [3]. From a hemodynamic perspective, preeclampsia is characterized by increased afterload, a normal or low left ventricular preload, a normal or reduced cardiac output, and impaired systolic and diastolic function [4]. Regarding the respiratory system, preeclampsia induces exaggerated capillary permeability, which can exacerbate airway edema, potentially making intubation significantly more challenging [5]. Additionally, severe tissue hypoxia associated with preeclampsia leads to an overproduction of lactate. At the same time, organ dysfunction impairs lactate metabolism and clearance, resulting in elevated circulating lactate levels [6]. In conclusion, preeclampsia appears to be a rapidly progressing and unpredictable multi-organ disease. It is primarily driven by endothelial damage, increased endothelial permeability, and tissue hypoxia, leading to multi-organ dysfunction in response to abnormal placental invasion, development, and function. Although the primary complications of preeclampsia include eclampsia, HELLP syndrome, and fetal growth restriction, severe cases may also result in pulmonary complications such as acute respiratory failure. Increased endothelial permeability, fluid overload, and cardiopulmonary compromise contribute to this life-threatening condition [7]. In this case report, we present a patient who developed peripartum respiratory failure in the setting of severe preeclampsia, requiring intensive care management.

## Case presentation

A 23-year-old primiparous woman at 36 weeks and 3 days of gestation presented to our emergency department with severe gestational hypertension (160/100 mmHg). She reported mild headaches and lower limb edema but denied visual disturbances or epigastric pain. Her oxygen saturation was 99%, and her pulse rate was 80 bpm. She had no significant medical history and was a non-smoker. Laboratory tests showed mild leukocytosis (9,540/μl), neutrophilia (67%), and a protein-to-creatinine ratio of 2.2 mg/dL (Table 1) [8].

**Table 1:** Laboratory tests at patient's admission SGOT: Serum glutamic-oxaloacetic transaminase; SGPT: serum glutamic pyruvic transaminase

Laboratory Tests	Value	Normal Range
WBC (White Blood Cell)	9540/μl	4-10 K/μl
Neutrophilia	67%	40-75%
Platelet Count	195.000	150-400 K/μl
Glucose	140 mg/dl	74-106 mg/dl
Creatinine	0.65 mg/dl	0.51-0.95 mg/dl
SGOT	28 U/l	<35 U/l
SGPT	14 U/l	<35 U/l
Uric Acid	6.8 mg/dl	2.6-6.6 mg/dl
Protein-to-Creatinine Ratio (Urine Sample)	2.2	<0.3

The patient was diagnosed with preeclampsia and admitted for close monitoring. Antihypertensive therapy with methyldopa (500 mg three times daily) was initiated. Fetal assessment, including ultrasound and cardiotocography, showed reassuring findings.

On the fourth day of hospitalization, despite antihypertensive treatment, her blood pressure remained elevated (155/110 mmHg), and she complained of worsening headaches. Due to a low Bishop score, an emergency caesarean section was performed instead of labor induction. Magnesium sulfate was administered for seizure prophylaxis [8].

During intubation, she developed transient bronchospasm with oxygen desaturation (SpO2 80%), which improved after intravenous adrenaline administration. However, hypoxemia persisted despite 100% FiO2. Arterial blood gas analysis (Table 2) revealed lactic acidosis and respiratory failure, prompting ICU transfer for specialized monitoring.

**Table 2:** Arterial blood gas test with FiO2 (fraction inspired oxygen) 100%

Arterial Blood Gas Test	Value	Normal Range
pH	7.21	7.35-7.45
PO_2_	106 mmHg	80-100 mmHg
PCO_2_	41 mmHg	35-45 mmHg
HCO_3_	16.5 mEq/L	22-26 mEq/L
Lactic Acid	3.47 mmol/L	<2mmol/L
BE (Base Excess)	10.7	

**Figure 1:**
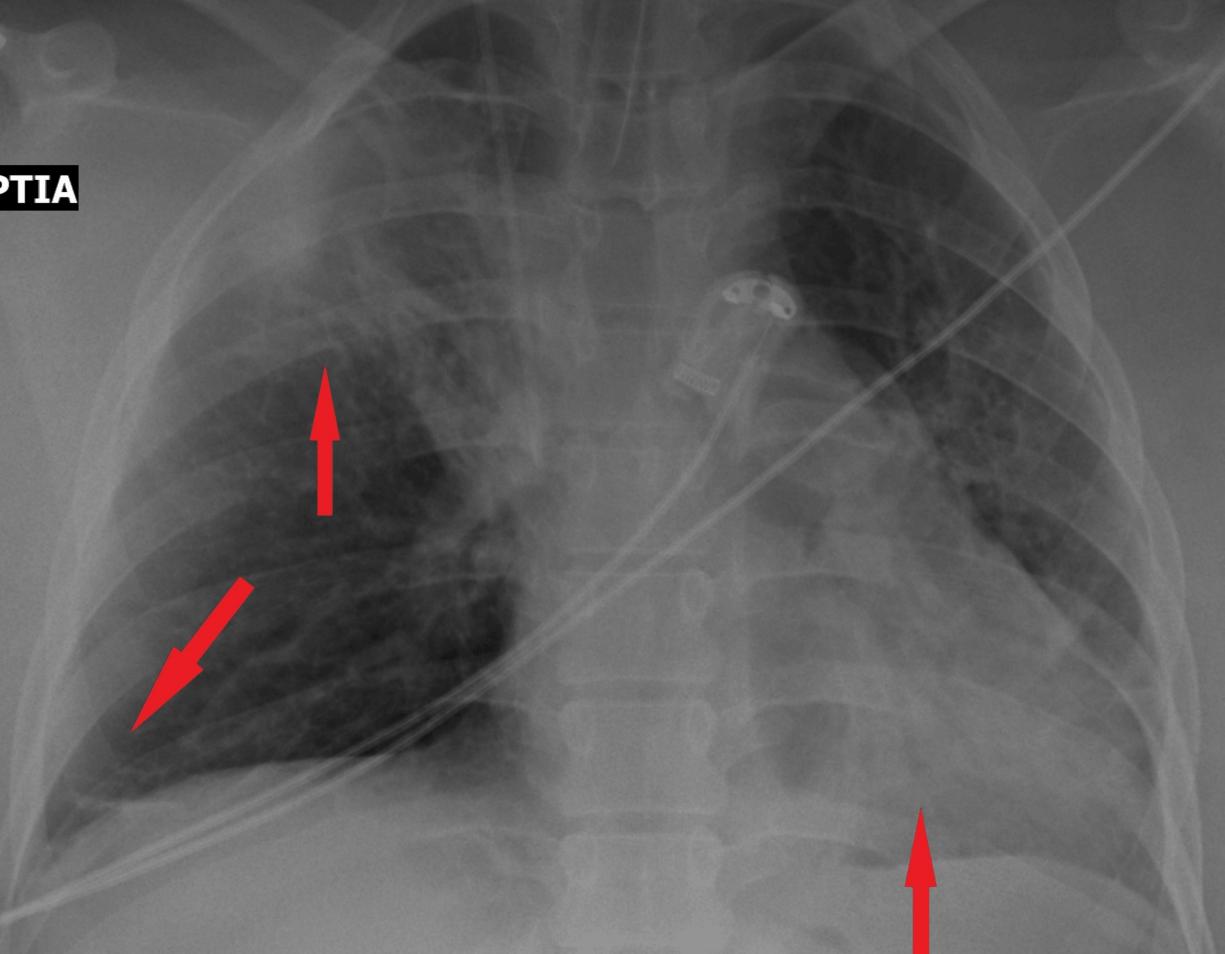
X-ray of chest with atelectasis of the upper right lobe and both lower lobes.

**Figure 2:**
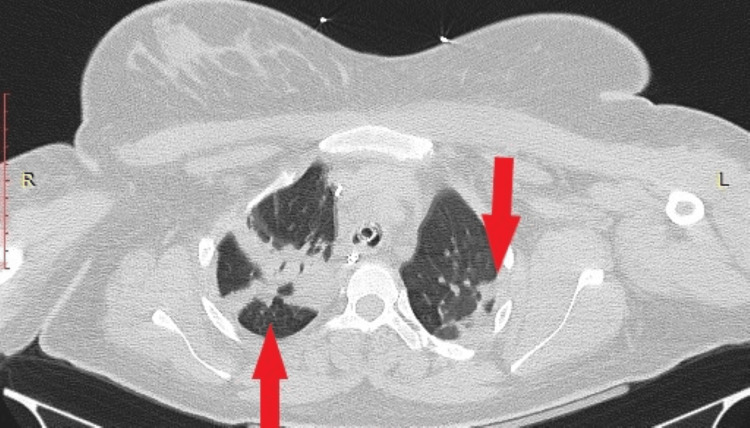
Computed tomography (CT) of chest in the axial plane with intravenous contrast showing atelectasis of both lower lobes.

Imaging (thorax X-ray and CT) ruled out cerebral edema, stroke, pulmonary embolism, pulmonary edema, infection, and ARDS with the only finding being lung atelectasis in the right upper and both lower lobes (Figures 1, 2).

Echocardiography showed preserved cardiac function with no signs of peripartum cardiomyopathy or patent foramen ovale. In greater detail, the echocardiogram demonstrated preserved overall left ventricular systolic function, with no evidence of segmental hypokinesia and adequate wall thickness. The ejection fraction (EF) was greater than 50% (normal range: 50-70%). The dimensions of both the left and right heart chambers were within normal limits, and the atrioventricular valves appeared structurally and functionally normal.

**Figure 3:**
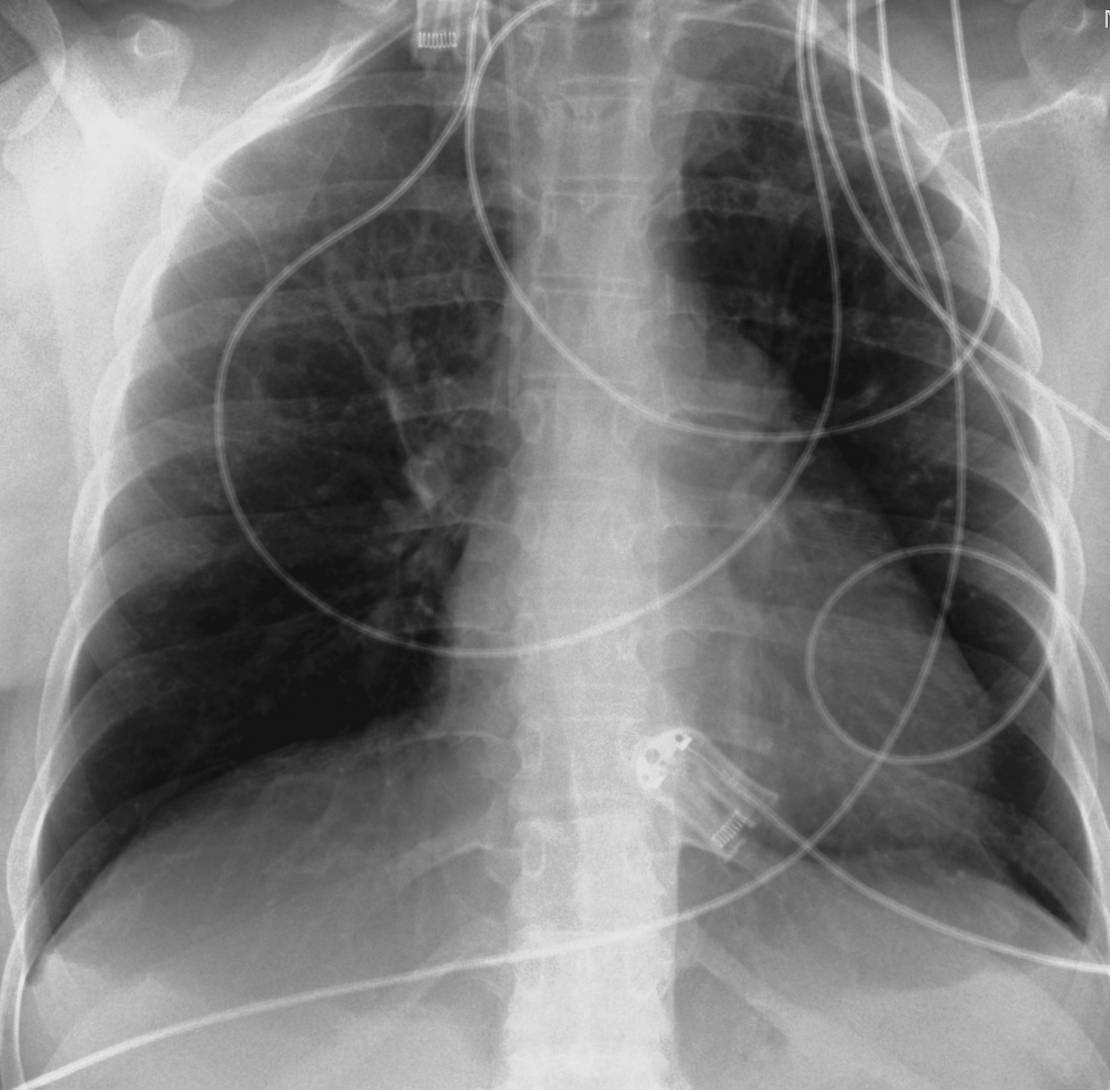
X-ray of chest on the day of discharge from the ICU (Intensive Care Unit) which was normal.

She was managed in the ICU for eight days, showing gradual respiratory improvement. Upon discharge from the ICU, she required 3 L/min nasal oxygen therapy and oral antihypertensive medication. A follow-up chest X-ray on the day of discharge showed resolution of atelectasis (Figure 3).

After two additional days in the Obstetrics Department, she was discharged in stable condition, with follow-up instructions for cardiology and pulmonology evaluations in 15 days. Three months later, the patient visited the outpatient clinic for a routine gynecological check-up. Cardiological and pulmonological follow-up, two months after the cesarean section, did not reveal any pathological findings, and there was no need for medication such as antihypertensives.

## Discussion

Acute respiratory failure is a rare but serious complication in pregnancy, affecting approximately 1 in 500 pregnancies [9]. It may arise from pregnancy-related disorders such as preeclampsia, amniotic fluid embolism, pulmonary edema, and peripartum cardiomyopathy or from non-pregnancy-related conditions such as asthma, respiratory infections, non-cardiogenic pulmonary edema, and pulmonary embolism [10].

In our patient, respiratory failure occurred during caesarean section. She had no prior respiratory disease, and intubation was uneventful. Transient bronchospasm resolved, yet persistent hypoxemia and elevated lactate levels suggested a respiratory impairment. Major complications were ruled out, leaving endothelial permeability, airway edema, and tissue hypoxia as potential contributing factors to her condition [11-13]. The exclusive imaging, finding that of pulmonary atelectasis, may result from the pressure exerted on the thorax during full-term pregnancy, increased body weight, and limited patient mobilization. Nevertheless, it definitely does not justify severe respiratory failure in a 23-year-old patient with no prior medical history. Despite preventive measures and close monitoring, delivery remains the only definitive treatment for preeclampsia. This case highlights the importance of early recognition, differential diagnosis, and management of complications such as respiratory failure in preeclampsia patients.

## Conclusions

Preeclampsia remains a significant cause of maternal morbidity and mortality, with potential complications affecting multiple organ systems. Acute respiratory failure, although rare, can be life-threatening and requires prompt recognition and intervention. This case highlights the importance of comprehensive monitoring, early diagnosis, and a multidisciplinary approach to optimize maternal and fetal outcomes.
